# Hutchinson-Gilford progeria syndrome complicated with stroke: A report of 2 cases and literature review

**DOI:** 10.3389/fped.2022.1056225

**Published:** 2022-11-29

**Authors:** Jingjing Wang, Qinmei Yu, Xiaohui Ma, Zhefeng Yuan, Jianhua Mao

**Affiliations:** ^1^ Department of Nephrology, Children’s Hospital, Zhejiang University School of Medicine, National Clinical Research Center for Child Health, Hangzhou, China; ^2^ Department of Radiology, Children’s Hospital, Zhejiang University School of Medicine, National Clinical Research Center for Child Health, Hangzhou, China; ^3^ Department of Neurology, Children’s Hospital, Zhejiang University School of Medicine, National Clinical Research Center for Child Health, Hangzhou, China

**Keywords:** HGPS, stroke, LMNA, clinical manifestations, imaging features

## Abstract

**Background:**

Hutchinson–Gilford Progeria Syndrome (HGPS) is a ultrarare, fatal autosomal dominant disorder. The pathogenesis of the disease is a mutation in *LMNA*, which leads to the accumulation of progerin in cells, impairing the normal physiological functions. Stroke and transient ischemic attack seriously affect the survival rate and quality of life of HGPS children, although the literature of this aspect is limited. This study summarizes the clinical manifestations and related imaging features of HGPS children with stroke to improve pediatric clinicians' understanding of this disease.

**Case presentation:**

Both children have a *de novo* heterozygous mutation of *LMNA* [c.1824C > T ( p.G608G)]. Case 1. At the age of 4 years, the child had a cerebral infarction, which manifested as blurred vision and communication disturbance. Multiple abnormal signals were observed on the head MRI in the bilateral frontoparietal cortex, bilateral semiovale center, lateral ventricle, and deep frontal and parietal lobes. Multiple abnormal white matter signals on head MRA: bilateral internal carotid artery stenosis with basilar artery, and bilateral thickening of the posterior communicating artery. Case 2. At the age of 8.5 years, the child presented with cerebral infarction, which manifested as decreased muscle strength and choking after drinking water. MRI of the head showed that the bilateral frontal lobes were small with multiple abnormal signal shadows in the bilateral center of the semiovale and the lateral ventricle. Brain MRA revealed that the bilateral internal carotid arteries (C5–7) were narrow and uneven in thickness, and the A1 segment of the left anterior cerebral artery was narrower than the contralateral one. After symptomatic and supportive treatment, the two children improved.

**Conclusion:**

Hemiplegia and physical weakness are the most prevalent stroke symptoms in children with HGPS, followed by headache, epilepsy, dysarthria, and psychosis as the primary manifestation in some children. Stroke in children with HGPS is mostly ischemic cerebral infarction caused by an insufficient cerebral blood supply. Pediatric cerebral infarction mainly occurs in the large vascular area, involving all vascular areas, with the internal carotid artery and middle cerebral artery being the most commonly accumulated.

## Introduction

Hutchinson–Gilford progeria syndrome (HGPS) is a ultrarare fatal syndrome of segmental premature aging that begins in the early childhood. It has an estimated overall incidence rate of 1 in 4–8 million newborns (1). HGPS arises from a sporadic autosomal dominant mutation in *LMNA*, which produces an aberrant form of the inner nuclear membrane protein lamin A known as progerin. Progerin is expressed in differentiated cell types, including vascular smooth muscle cells, endothelial cells, and adventitial fibroblasts, which are integral to the vascular structure and function. HGPS is manifested as growth retardation, a gradual progression of characteristic facial features, the loss of hair and subcutaneous fat, restricted joint mobility, and rapidly progressing severe atherosclerosis. Children with HGPS appear superficially normal at birth, but they start developing substantial growth delays within the first year of life. Subsequently, their symptoms worsen with time. The afflicted children have a median life expectancy of 14.6 years (2), and the most common causes of death are cardiovascular complications such as stroke or myocardial infarction. Despite the extremely low prevalence of stroke in HGPS, little is known about the nature of strokes and the vascular characteristics of the head of these patients. Herein we have reported the cases of two HGPS patients who experienced strokes. This study analyzed the clinical and imaging characteristics of HGPS children with stroke in conjunction with the relevant literature review to improve the pediatriciansâ€™ knowledge of this disease.

### Case presentation

#### Case 1

A 4-year-old boy patient was admitted to the hospital with the chief complaint of “hard skin for more than 3 years, unclear vision, and communication obstacle for a day” with no family history of HGPS. The boy appeared normal at birth, weighing 3 kg, but he soon failed to thrive. After 1 month of birth, his skin appeared swollen and sclerodermatous on the trunk, with the loss of scalp hair along with stunted growth. Before his admission, the child experienced blurred vision, followed by communication difficulties, dull eyes, and an inability to speak and communicate.

**Figure 1:**
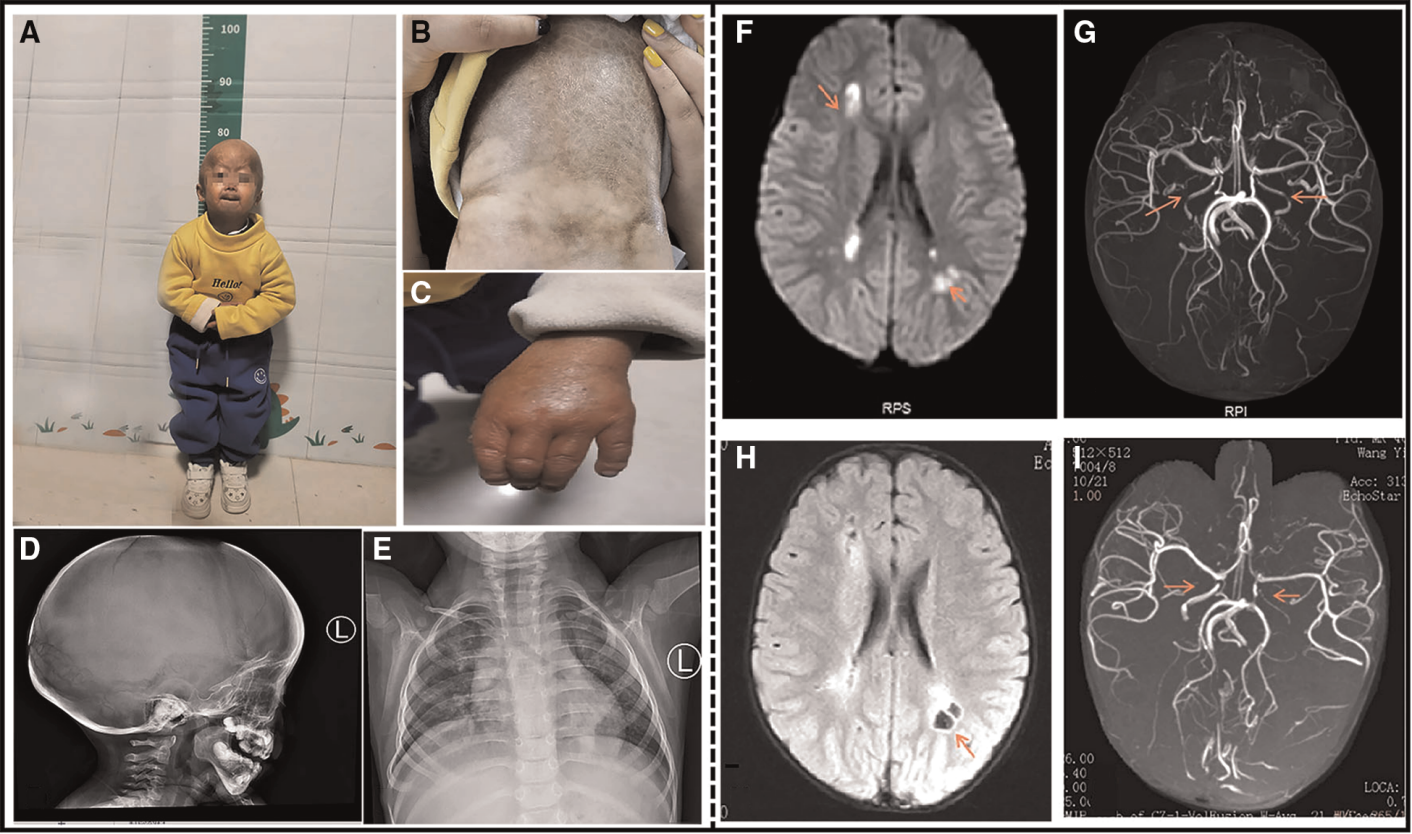
A 4-year-old boy patient. (**A**) The child has severe growth retardation; he showed distinctive facial features, scalp veins, generalized alopecia with sparse downy hairs, narrow nasal bridge, thin lips, and micrognathia. (**B**) Dry and stiff skin on the entire body, poor elasticity, and a lot of mottled pigmentation on the trunk. (**C**) Flexion deformity of the proximal phalangeal joints of both the fingers. (**D**) The shape of the head is enlarged, the anterior fontanelle and cranial suture are not closed, and the cranial plate of the parietal occipital region is thinned. (**E**) Multiple deformities of the ribs on both sides, dysplasia of the clavicle and scapula on both sides. (**F**) Cranial MRI at the time of onset: Multiple abnormal signals around the lateral ventricle. (**G**) Cranial MRA at the time of onset: The red arrow indicates bilateral internal carotid artery stenosis. (**H**) After the 3-month review, Cranial MRI: bilateral paraventricular lacunar infarction. (**I**) After the 3-month review, Cranial MRA: The red arrow indicates bilateral internal carotid artery stenosis similar to that noted earlier.

On examination, the child showed distinctive facial features, with prominent eyes and scalp veins, delayed closure of the anterior fontanelle, generalized alopecia with sparse downy hairs, narrow nasal bridge, thin lips, micrognathia, and small ear lobes (Figure 1A). His neck is soft, Brinell's sign is negative, Klinefelter's sign is negative, Babbitt's sign is positive on both sides, tendon reflexes are present, limb muscle strength is grade IV, muscle tension is high , and the skin on the entire body is stiff with poor elasticity, and a lot of mottled pigmentation is present on the trunk (Figure 1B). Bilateral metacarpophalangeal joint contracture deformity (Figure 1C), lexion deformity of both the proximal knuckles, squatting difficulty, and hip and knee joint contractures were fixed, and a horse-riding posture was detected. He was 77 cm tall (<1st percentile) and weighed 7.8 kg (<1st percentile). He could not pronounce and communicate, and he was too young to receive detailed ophthalmic examination.

The complete blood count, liver and renal function tests, cardiac enzymes, serum electrolytes, and lipids were within the normal limits. An electrocardiogram and ultrasonic cardiogram revealed no anomalies. His radiographic examination revealed general osteoporotic changes, with short and dystrophic clavicles and distal phalanges osteolysis. His lateral skull X-ray revealed cranial dysplasia (Figure 1D). Chest X-ray showed bilateral clavicle dysplasia and bilateral rib deformity (Figure 1E). Carotid artery B-ultrasound revealed 0.05 cm intima-media thickening in both common carotid arteries. EEG revealed moderate abnormalities, with more irregular delta waves. Brain MRI revealed multiple abnormal signals in the bilateral frontoparietal cortex, bilateral semiovale center, and lateral ventricle, as well as multiple abnormal signals in the deep frontoparietal white matter, suggesting cerebral infarction (Figure 1F). His cranial MRA revealed bilateral internal carotid artery stenosis with basilar artery and thickening of the bilateral posterior communicating artery (Figure 1G). A re-examination of his head MRI after 3 months revealed bilateral paraventricular and central semiovale lacunar infarction (Figure 1H). A re-examination after 3 months of head MRA bilateral internal carotid artery was similar to the previous one (Figure 1I). The patient's history and examination findings supported the diagnosis of HGPS. DNA sequence analysis was performed to confirm the clinical diagnosis. The patient carried a c.1824C > T (p.G608G) heterozygous *LMNA* mutation.

There is no specific treatment plan for this child. After admission, cerebroside carnosine was used to protect the brain, nimodipine to dilate the blood vessels, low molecular weight heparin combined with aspirin for anticoagulation, low molecular weight dextran to improve circulation, and mannitol to reduce intracranial pressure.

The patient's consciousness improved after treatment and he was discharged from the hospital, with a 1-month follow-up scheduled at the neurology department. The child was conscious, could pronounce and communicate normally, and could walk on his own. Three months after discharge from the hospital, his head MRI revealed bilateral paraventricular and central semiovale lacunar infarction, while his head MRA showed bilateral internal carotid artery stenosis.

#### Case 2

The second case is an 8.5-year-old boy who was born at term through cesarean section owing to “low fetal movement and cloudy amniotic fluid”. His birth weight was 3.1 kg, and he had a normal family history. He was admitted to our hospital for “sclerosis of the skin for more than 8 years, decreased muscle strength of the right limb, choking on drinking water for 3 days, and vomiting for a day”. Similar to Case 1, the child developed progressive skin sclerosis approximately 20 days after delivery, after which he gradually developed hair loss, misaligned teeth, and joint deformities, accompanied by severe growth retardation. Notably, the child developed recurrent paroxysmal headaches at the age of 3 years, with no obvious abnormality in the head MRI; as a result, no treatment was prescribed at this time. Three months before his admission, the child developed severe headache that improved after oral administration of aspirin. Three days before his admission, he developed weakness in his right upper extremity and choked easily on drinking water, followed by vomiting.

**Figure 2:**
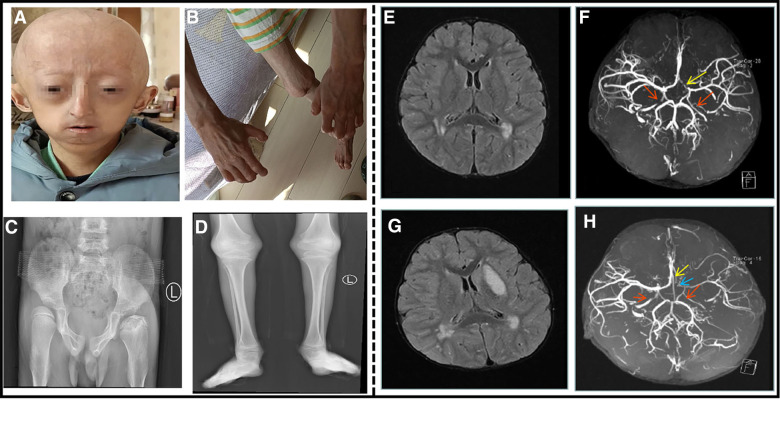
An 8.5-year-old boy patient. (**A**) The child showed distinctive facial features, with prominent forehead, baldness, exposed scalp veins, long and narrow nose, thin upper lip, and micrognathia. (**B**) The child exhibited flexion deformity of the distal knuckle joints of both the fingers, abduction of the bilateral wrist. (**C**) Anteroposterior pelvis X-ray showed bilateral hip dysplasia, bilateral femoral heads laterally positioned and left femoral head aseptic necrosis. (**D**) Long bones with thin diaphysis, widened metaphysis, and decreased bone density. (**E**) Cranial MRI at the time of onset: multiple abnormal signal shadows in the lateral ventricle. (**F**) Cranial MRA at the time of onset: bilateral internal carotid artery stenosis. The red arrow indicates internal carotid artery stenosis, and the yellow arrow indicates left anterior cerebral artery stenosis. (**G**) After the 3-month review, cranial MRI: multiple abnormal signal shadows in the lateral ventricle, and the left basal Nodal lesions. (**H**) After the 3-month review, cranial MRA: his left internal carotid artery, left middle cerebral artery and left cerebral artery had stenosis. The red arrow indicates internal carotid artery stenosis, the blue arrow indicates left middle cerebral artery stenosis, and the yellow arrow indicates left anterior cerebral artery stenosis.

Physical examination: Similar to case 1, there is progeria appearance, which is manifested as a prominent forehead, baldness, exposed scalp veins, long and narrow nose, thin upper lip, micrognathia, and irregular dentition (Figure 2A). Neurological examination showed a soft neck, a negative Brucella sign, a negative Klinefelter sign, a positive bilateral Barthel sign, tendon reflexes, and right upper extremity muscle strength grade IV. The child exhibited flexion deformity of the distal knuckle joints of both the fingers (Figure 2B), abduction of the bilateral wrist and hip joints, flexion of the bilateral wrist and ankle joints, and anteversion of the spine. The child has a stiff, dry, and poorly elastic, with a lot of mottled pigmentation on the torso.

His complete blood count, liver and renal function tests, cardiac enzymes, serum electrolytes, and lipids were within normal limits. An electrocardiogram and ultrasonic cardiogram revealed no anomalies. Chest X-ray showed bilateral clavicle dysplasia and bilateral rib deformity; anteroposterior pelvis X-ray showed bilateral hip dysplasia, bilateral femoral heads laterally positioned and left femoral head aseptic necrosis (Figure 2C); and the long bones of his limbs were thin and dry (Figure 2D). The epiphysis was relatively wide, and the bilateral elbow angle was negative (cubitus varus); the lateral X-ray of the skull revealed cranial dysplasia. Carotid artery B-ultrasound showed that the bilateral carotid intima was slightly thickened (about 0.1 cm), and the bilateral internal carotid lumen was locally narrowed. The EEG was normal, and his head MRI revealed that the bilateral frontal lobes were small, with multiple abnormal signal shadows in the bilateral center of the semiovale and the lateral ventricle (Figure 2E). His brain MRA indicated that the bilateral internal carotid arteries (C5–7) were narrow and uneven in thickness, and the A1 segment of the left anterior cerebral artery was narrower than the contralateral one (Figure 2F). After the 3-month review, his head MRI revealed multiple abnormal signal shadows in the two cerebral hemispheres indicating an ischemic foci, the left basal Nodal lesions, and cerebral infarction to be discharged (Figure 2G). At re-examination after 3 months, his brain MRA revealed stenosis of his left internal carotid artery, left middle cerebral artery and left cerebral artery (Figure 2H). The proximal anterior artery was narrowed as was the right internal carotid artery. Genetic testing revealed a *de novo* heterozygous mutation in the chromosome 1 *LMNA* (c.1824C > T (p.G608G)). After admission, he was administered enalapril maleate, valsartan, amlodipine besylate to lower blood pressure, cerebroside carnosine to protect the brain, nimodipine to dilate the blood vessels, and low molecular weight heparin mixed with aspirin for anticoagulation. He was treated with glycosides to improve circulation and mannitol to lower the intracranial pressure before discharge from the hospital. After discharging from the hospital, his rehabilitation training was continued. After 2 months later, the muscle strength of his right upper extremity basically returned to normal and he no longer choked after drinking water.

## Discussion

HGPS is a ultrarare, fatal, premature aging syndrome. Most HGPS infants appear healthy at birth, but they begin to exhibit aging features within the first year of life (3). The most common early symptoms of HGPS are skin tightness or bulge in the abdomen and/or thigh area, while the other symptoms include growth retardation, loss of body fat, and hair loss skin changes (4), hip dislocation (5), generalized atherosclerosis, cardiovascular disease, and stroke. However, most intellectual development is normal, unless there is a sequelae caused by stroke (6). With an average age of 14.6 years, the most common causes of death among children with HGPS are cardiovascular complications such as stroke or myocardial infarction. Individuals with classic genotype HGPS are heterozygous for pathogenic variant c.1824C > T (∼90% of individuals with HGPS). The *LMNA* c.1824C > T mutation results in the activation of a cryptic donor splice site leading to the production of a lamin A isoform, called progerin, which contains an internal deletion of 50 amino acids near its C-terminal end. This deletion eliminates the site for endo-proteolytic cleavage by ZMPSTE24, a zinc metalloproteinase that cleaves the 15 C-terminal amino acids of prelamin A to yield mature lamin A. Therefore, progerin retains its farnesyl moiety at the C-terminus. Retention of the farnesyl group causes progerin to become permanently anchored into the nuclear membrane, leading to a whole variety of abnormalities in the nuclear processes, which eventually lead to cellular and organismal decline (7). Individuals with nonclassical genotype HGPS show the characteristic clinical features of HGPS and are heterozygous for another LMNA pathogenic variant in exon 11 or intron 11, which results in the production of progerin (∼10% of individuals with HGPS) (8, 9). Until now, a total of 11 *LMNA* pathogenic variant in the nonclassical genotype HGPS have been identified, and their clinical phenotypes vary in severity (see details in Table 1).

**Table 1:** Genotypes of HGPS, Causative LMNA Variants and Clinical Phenotypes.

Genotype	DNA Mutation	Amino Acid Effect	Phenotypic Features Compared to Classical HGPS	Reference
Classical HGPS	1824 C > T, exon 11	G608G	See Footnote^*^	(8)
	1822 G > A, exon 11	G608S	Moderate	(8)
	1821 G > A, exon 11	V607V	Severe; neonatal progeria	(18)
	1868 C > G, exon 11	T623S	Mild	(19)
	1940 C > T, exon 11	L647A	Very mild	(20)
	1968 G > A, exon 11	Q656Q	Very mild	(21)
Non-classical HGPS	1968 + 1 G > C, intron 11	------	Severe	(9)
	1968 + 1 G > A, intron 11	------	Severe	(22)
	1968 + 2 T > A, intron 11	------	Mild	(23)
	1968 + 2 T > C, intron 11	------	Mild	(9)
	1968 + 5 G > A, intron 11	------	Very mild	(21)
	1968 + 5 G > C, intron 11	------	Moderate	(9)

^*^ Clinical features of HGPS: Severe failure to thrive, Progressive alopecia, Skin lesions, Characteristic facies, Loss of subcutaneous fat, Bone changes, Skeletal anomalies, Musculoskeletal degeneration, Hearing loss, High-pitched voice, Delayed and crowded dentition, Atherosclerosis, Cerebrovascular disease, and others.

The two case reports discussed in this study showed no obvious abnormalities at birth, and both started developing progressive hardening of the skin around 1 month after birth, along with severe growth retardation, hair loss, irregular teeth, joint deformities, and other typical premature aging symptoms. Both the children are classic genotype HGPS. Children usually have a lower stroke incidence than adults, and the most common risk factors include infection and trauma (10). For children with HGPS, progerin produced by *LMNA* mutation is deposited in the blood vessels, causing premature aging and disappearance of smooth muscle cells, resulting in fibrosis, atheromatous changes, and hardening of the blood vessels (11), especially those of the head and neck. To compensate, the blood vessels form the auxiliary vessels or “bypasses,” to aid the blood flow and supply oxygen to the areas of the brain that were previously served by narrowed arteries. However, these new blood vessels are smaller and more fragile than normal blood vessels, which increases the risk of stroke. The patients in this study showed no symptoms of infection such as fever, cough, diarrhea, and no clear history of trauma; therefore, the stroke was considered to result from the primary disease.

Cerebrovascular events can occur in children with HGPS at any age. The first symptom is usually stroke or transient ischemic attack, which seriously affects the quality of life in children. The earliest report in the literature shows the age of occurrence of 4 years, which is similar to that of case 1 in this report. The symptoms are also comparable with those associated with other secondary causes of stroke in children, such as embolism and cerebrovascular malformation (12). We have summarized the clinical symptoms of children with HGPS complicated with stroke, indicating hemiplegia and physical weakness as the most prevalent stroke symptoms in children with HGPS, followed by headache, epilepsy, dysarthria, and psychosis as the primary manifestation in some children (Table 2). In this study, case 1 had a sudden blurred vision as the primary symptom, followed by communication disorder, dull eyes, inability to speak and communicate, and no other neurological symptoms such as hemiplegia and epilepsy. Case 2 was mainly manifested by decreased muscle strength of the right limb and choking after drinking water. Unlike for case 1, this child had a history of recurring headaches before the stroke. In addition, among the 10 cases we have counted, 1 child showed no symptoms at the time of cerebral infarction. However, according to Silvera et al, more than half of the HGPS children with clear imaging evidence of cerebral infarction do not have evident clinical symptoms (13). As a result, even in the absence of neurological symptoms, the children must be closely monitored with brain MRI and head and neck MRA examinations to determine the existence and severity of the intracranial diseases.

**Table 2:** Related reports of HGP children with stroke.

Case	Age at diagnosis of stroke (year)	Gender	Gene	Symptoms								Cranial MRI	Follow-up observation
				Headache	Hemiplegia	Epilepsy	Transient ischemic attack	Limb weakness	Dysarthria	Mental disorder	No clinical symptoms		
1(24)	5.5	Male	unknown	none	Yes	none	none	Yes	Yes	none	none	Multiple cerebral infarctions in the left cerebral hemisphere, temporal lobe, and basal ganglia (middle cerebral artery area)	Follow-up after 2 weeks: symptoms were basically relieved
2(25)	4.5	Female	unknown	none	none	none	none	None	none	Yes	none	Brain atrophy	Oral trifluoperazine for 3 months: symptoms improved.
3(26)	8	Female	unknown	Yes	Yes	none	none	Yes	none	none	none	Right superior parietal cortical infarction (upper-right middle cerebral artery)	Follow-up after 8 months: hemiplegia improved
4(27)	5	Male	unknown	none	Yes	Yes	none	None	Yes	none	none	Left periventricular white matter infarction; (watershed infarction between right and left middle cerebral artery and posterior cerebral artery regions; left anterior frontal cerebral artery region infarction)	Follow-up after 3 months: multiple seizures, no new infarction was found on MRI; Follow-up after 5 months: seizures, new left temporoparietal infarction; Follow-up after 8 months: seizures under control after gabapentin
5(28)	4	Male	unknown	Yes	Yes	Yes	Yes	Yes	none	none	none	Subdural effusion in both frontal and posterior parietal lobes, diffuse periventricular white and basal ganglia ischemic disease, and right posterior parietal infarction (anterior cerebral artery)	Follow-up after 6 months: hemiplegia improved compared with before, there was transient limb weakness, and brain atrophy gradually appeared
6(29)	9	Male	*LMNA* c.1824 C>T(p. G608 G)	Yes	Yes	none	Yes	Yes	none	none	none	Bilateral small frontal lobe infarction, and right parietal lobe large infarction (anterior cerebral artery)	Follow-up after 2 and 6 months: transient ischemic attack; Follow-up at age 10: sudden death
7(30)	6	Male	unknown	none	none	none	none	None	none	none	Yes	Right putamen infarction (anterior cerebral artery)	No follow-up records
8(31)	4.5	Male	unknown	none	Yes	none	Yes	None	none	none	none	Severe ischemic changes in the basal ganglia and subcortical ischemic changes in the right frontal lobe (anterior cerebral artery)	Follow-up at age 7: death from hemorrhagic stroke
9	4	Male	*LMNA* c.1824 C>T(p. G608 G)	none	none	Yes	none	None	Yes	none	none	Multiple abnormal signals in bilateral fronto-parietal cortex, bilateral centrum semiovale and periventricular (bilateral internal carotid artery stenosis)	Follow-up after 2 month: symptoms are basically relieved
10	8.5	Male	*LMNA* c.1824 C>T(p. G608 G)	Yes	Yes	none	Yes	Yes	none	none	none	Multiple abnormal signal shadows in both cerebral hemispheres, left basal ganglia lesions (the bilateral internal carotid arteries (C5-7))	Follow-up after 3month: symptoms are basically relieved

In this study, we collected relevant reports on HGPS children with stroke over the years. The results showed that most strokes in children with HGPS are ischemic cerebral infarction caused by the insufficient cerebral blood supply. However, children can die from cerebral hemorrhage secondary to ischemic cerebral infarction. In children, the main cerebral infarction occurs in the field of large blood vessels, which involves all vascular regions, and mostly affects the internal carotid artery, anterior and middle cerebral arteries, and posterior artery stenosis is rare (Table 2). Children with HGPS often suffer from chronic brain hypoperfusion, which can easily cause ischemic damage to the cerebral white matter. Confluent white matter lesions in the cerebral hemisphere are also common (14). Multiple abnormal signals were seen in the bilateral frontoparietal cortex and around the lateral ventricle in Case 1, along with multiple abnormal signals in the deep frontal and parietal white matter. In Case 2, multiple abnormal signal shadows appeared in the child's bilateral cerebral hemispheres, all of which had such characteristics.

There is currently no specific treatment for HGPS. The main treatment is to relieve symptoms and control secondary organ dysfunction. Lonafanib, an orally active farnesyl protein transferase (FPTase) inhibitor, has been approved by the US FDA for the treatment of HGPS and some specific progeroid laminopathy. It can effectively reduce the possibility of heart disease and stroke in children with HGPS and prolong the life of HGPS children, but it cannot reverse and prevent the progression of the disease (15). Moreover, it is very expensive and has not been on market in China.

Treatment of atherosclerosis, stroke and other complications is achieved through standard conventional monitoring and medical therapy, but the disorder will continue to progress. Low-dose aspirin is currently recommended for preventing heart disease and stroke in children with HGPS. Moreover, since hardened vascular systems are more susceptible to dehydration, oral intake is recommended for optimal hydration. Appropriate physical activity is also encouraged to minimize stroke risk. Once a child is suspected of having a stroke, symptomatic and supportive care should be given as soon as possible: 1) oxygen supplementation and fluid infusion should be applied to improve their vascular status; 2) anticoagulants other than the routinely recommended aspirin may be warranted, such as low molecular weight heparin, warfarin, rivaroxaban and others (9); 3) controlling blood pressure and epileptic seizures is also important to reduce reversible and irreversible brain damage due to cerebral infarction. In this study, both children administered cerebroside carnosine to protect the brain, nimodipine to dilate the blood vessels, low molecular weight heparin mixed with aspirin for anticoagulation, glycosides to improve circulation and mannitol to lower the intracranial pressure. After treatment, the symptoms of the two children improved significantly.

Interestingly, the brain MRI features of these HGPS children with stroke resemble early stages of moyamoya vasculopathy which is also a steno-occlusive disease of the cerebral arteries, so in addition to antiplatelet, revascularization is a potential preventive approach (16, 17). However, due to the poor prognosis of HGPS, revascularization was not an option for stroke prevention in either case. There is no report on thrombolysis or revascularization in children with HGPS complicated with cerebral infarction.

## Data Availability

The original contributions presented in the study are included in the article/Supplementary Materials, further inquiries can be directed to the corresponding author/s.
